# Pregnancy of Unknown Location: The Value of Frozen Section Analysis and Its Relation to Beta-hCG Levels and Endometrial Thickness

**DOI:** 10.1055/s-0038-1676123

**Published:** 2018-12-12

**Authors:** Marwan Odeh, Ayat Qasoum, Rene Tendler, Mohamad Kais, Rola Khamise Farah, Jacob Bornstein

**Affiliations:** 1 Department of Obstetrics and Gynecology, Galilee Medical Center, Nahariya, Israel; 2 Azrieli Galilee Faculty of Medicine, Bar Ilan University, Safed, Israel

**Keywords:** ectopic pregnancy, frozen section, pregnancy of unknown location, β-hCG, endometrial thickness

## Abstract

**Objective** Frozen section examination is a rapid method for identifying products of conception in endometrial curetting, yet its accuracy is inconclusive. The purposes of this study is to determine the accuracy of frozen section analysis of endometrial curetting in pregnancies of unknown location, and to verify the relation of β-human chorionic gonadotrophin (hCG) level and endometrial thickness to the assessed accuracy.

**Methods** We reviewed data from January 2009 to December 2014 of diagnostic curettages from women with suspected ectopic pregnancies sent for frozen section examination at a medical center. A frozen section diagnosis was considered accurate if it concurred with the final pathologic diagnosis.

**Results** Of 106 frozen section studies, the diagnosis was accurate in 94 (88.7%). Of 79 specimens interpreted as negative on frozen sections (no products of conception noted), 9 (11.4%) were positive on final pathologic review. Three of the 27 (11.1%) specimens interpreted as positive by a frozen section failed to demonstrate products of conception on a final pathologic section. The sensitivity of frozen sections in the diagnosis of ectopic pregnancy was 72.7%, specificity 95.9%, positive predictive value 88.9%, negative predictive value 88.6%, and accuracy 88.6%. A statically significant correlation was found between β-hCG level and high accuracy of the frozen section technique (*p* < 0.001). No correlation was found between endometrial thickness and the accuracy of the frozen section technique.

**Conclusion** The accuracy of frozen section examination was high and was found to correlate with β-hCG level, but not with endometrial thickness.

## Introduction

Ectopic pregnancy can be identified early by the widespread use of transvaginal ultrasonography with high frequency probes, together with the use of sensitive quantitative measurements of serum human chorionic gonadotropin (hCG).^1^ ^2^ ^3^ ^4^ ^5^ ^6^ ^7^ Confirmation of the presence of intrauterine pregnancy by transvaginal ultrasonography is a highly accurate method of exclusion of an ectopic pregnancy, and in most cases has replaced diagnostic curettage as a cornerstone for ruling out or ruling in an ectopic pregnancy.^8^ However, in certain situations ultrasonography might result in diagnostic uncertainty, in which case an endometrial biopsy may still be necessary to distinguish between abnormal intrauterine and ectopic pregnancies.

Frozen section evaluation of uterine curetting is performed in certain instances of pregnancy of unknown location (PUL), to rule out ectopic pregnancy. If chorionic villi are identified *in utero*, an ectopic gestation is essentially ruled out (except for rare cases of heterotopic pregnancies) and no further treatment is needed. If villi are not detected, this may reflect an ectopic pregnancy, although the patient may also have expelled all intrauterine villi. The absence of chorionic villi on endometrial frozen section may lead to laparoscopy, laparotomy, salpingectomy or methotrexate therapy, despite the fact that an ectopic pregnancy is not always present.^8^ Therefore, diagnosis using the frozen section technique performed on endometrial material shortly after curettage and after conception products have been identified can dramatically decrease the time needed to rule out the presence of an ectopic pregnancy. Such diagnosis also avoids the undesired administration of methotrexate to women with intrauterine pregnancy.

The accuracy of endometrial biopsies using the frozen section technique has not been thoroughly evaluated. No systematic reviews have been conducted on this topic.

We found three relevant articles in PubMed and MedLine databases. Two of them highly recommended the use of frozen section examination as an accurate method for identifying products of conception in endometrial curetting. In their analysis of 87 cases in which frozen section assessment of an endometrial curettage specimen was performed, Spandorfer et al^9^ found that 81 (93.1%) were accurate. Only one woman was misdiagnosed and did not have an ectopic pregnancy. In a study conducted at our medical center, frozen section analysis had a sensitivity of 78%, specificity of 98%, positive predictive value of 95% and a negative predictive value of 93%.^10^ In that study of 70 frozen sections, 63 (90%) were accurate. Of 50 specimens interpreted as negative on frozen sections, 6 (12%) contained conception products on final pathologic review. One of the 20 (5%) specimens interpreted as positive by a frozen section failed to demonstrate products of conception on a final pathologic section. The sensitivity of frozen sections in the diagnosis of ectopic pregnancy was 76%; specificity, 98%; positive predictive value, 95%; negative predictive value, 88%, and accuracy, 90%. The accuracy of frozen section diagnoses was analyzed and stratified by preoperative serum hCG concentration. The cut-off point used for serum hCG was 1,000 IU/l.^10^ The percentage of inaccurate frozen section diagnoses was greater in the subgroup with higher serum hCG values (12.5%) than in the subgroup with lower values (9.3%). However, the difference between the two groups was not statistically significant.

In contrast to these two studies, Heller et al^11^ concluded that frozen section evaluation of uterine curetting can produce false negative diagnosis, and that this should be considered in the operative planning of women with suspected ectopic pregnancy. They reviewed 36 cases, of which 13 showed evidence of intrauterine pregnancy on final permanent sections. Five false negatives were identified in which no villi had been identified on a frozen section, but villi or evidence of an implantation site had been noted on final pathologic sections. The sensitivity of frozen sections in the diagnosis of ectopic pregnancy was 62%.

The purpose of the current study was to determine the accuracy of frozen section analysis in cases of pregnancy of unknown location, and the relation of this assessment of accuracy to β-hCG level and endometrial thickness.

## Methods

We performed a retrospective analysis of the Department of Obstetrics and Gynecology database in Galilee Medical Center from January 2009 to December 2014. In women with PUL, a diagnostic curettage was performed and the material from the curetting was sent for frozen section examination. PUL was defined as the situation of a positive pregnancy test with no signs of intra- or extrauterine pregnancy on transvaginal sonography.^12^ Curettage was performed only in cases with a plateau of β-hCG levels in at least two consecutive examinations; or β-hCG of more than 2,000 mIU/ml in 2 sequential measurements, 48 hours apart, a rise of less than 1.66 in β levels (48 h/0 < 1.66) and no evidence of intra or extrauterine pregnancy.

In the department of pathology, frozen sections were prepared from endometrial curetting, and blood clots were separated from the specimen macroscopically before freezing. The tissue was embedded in optimal cutting temperature aqueous medium and then it was frozen in liquid isopentane at -25°C. The frozen tissue was cut on a cryostat, and 5 to 9 μm sections of tissue were transferred to a glass slide at room temperature, stained with hematoxylin and eosin, and a cover slip was applied. The remaining frozen material was thawed and processed for routine paraffin fixation. Cases with negative result for chorionic villi have the samples supplied with β hCG, Vimentin and Pan Keratin A1/A3 immunohistochemical staining (Zymed#, Zymed Laboratories Inc., San Francisco, CA, USA). The entire frozen section process took no longer than 20 minutes, including microscopic inspection of the section.

The main variables investigated were frozen section diagnosis and final pathologic diagnosis, both variables according to the presence or absence of products of conception. Frozen section diagnosis was considered accurate if it concurred with the final pathologic diagnosis. Clinical and epidemiological data were also investigated, including age, week of gestation, β-hCG level, and endometrial thickness before the curettage took place.

Quantitative data, such as age, week of gestation, β-hCG level, and endometrial thickness, are described by mean and standard deviation, and by median and range. Qualitative data are presented as frequencies and percentages. This includes calculations of accuracy, sensitivity, specificity, and positive and negative predictive values. Accuracy, which was assessed as the concurrence or discrepancy between frozen section diagnosis and final pathologic diagnosis, was calculated for subgroups according to-β hCG level determined by receiver operator characteristic (ROC) curves. The cut-off point used was a serum hCG level of 1,000 IU/l. This level was chosen based on the ‘discriminatory zone’ values reported in the literature. This concept was developed by Kadar et al^13^ to determine the serum hCG level at which a sac should be seen on ultrasound examination. According to this concept, if the serum hCG level is above the ‘discriminatory zone’ and an intrauterine gestational sac is not seen, the pregnancy is abnormal, has aborted, or is in an ectopic location. Using vaginal sonography to detect the sac, the range of ‘discriminatory zones’ reported extends from 600 to 1,025 mIU/ml.^14^ ^15^ ^16^ ^17^

The t-test was used to examine the relation between the accuracy of frozen section and both β hCG level and endometrial thickness. A statistically significant result was defined if *p* < 0.05. The study was approved by the Institutional Review Board (Helsinki Committee) of Galilee Medical Center.

## Results

**Fig. 1:**
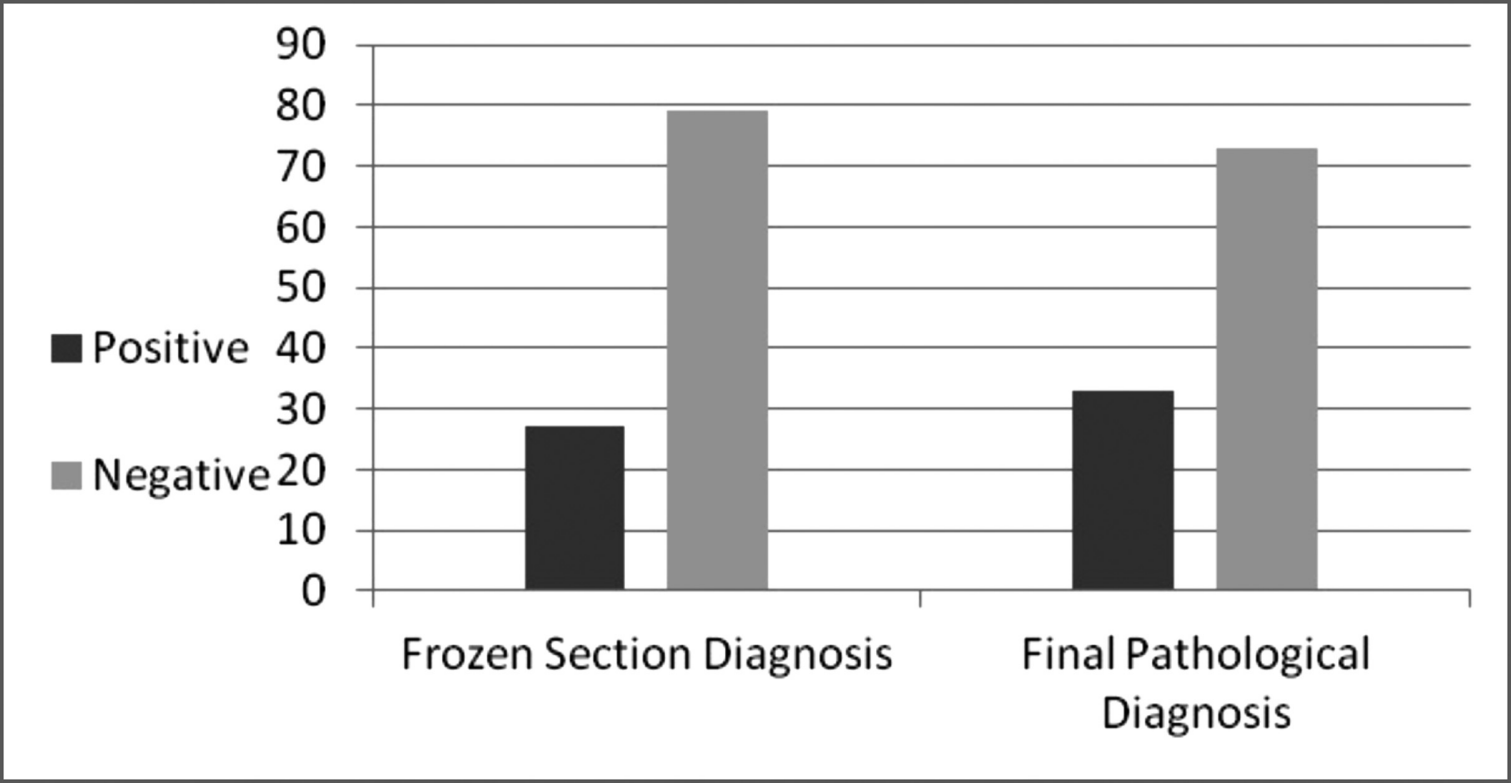
Results of frozen section and final pathological examination.

Data of 106 women were analyzed. Table 1 presents demographic and clinical data. Frozen sections were positive for products of conception in 27 (26%), whereas final pathological examination was positive in 33 (31%) (Fig. 1). The accuracy of frozen section analysis in detecting products of conception, when compared with the final pathological diagnosis, was 88.7%. The correlation between frozen section and final pathological diagnosis was statistically significant (*p* < 0.001), with phi = 0.729 (high strength). Of the 106 frozen section studies the diagnosis was accurate in 94 (88.7%) and inaccurate in 12 (11.3%). Of the 79 specimens interpreted as negative on frozen sections (no products of conception noted), 9 (11.4%) were found to contain conception products on final pathologic review. Three of the 27 (11.1%) specimens interpreted as positive by a frozen section failed to demonstrate products of conception on a final pathologic section. All three women were not discharged and received followed-up because of the low percentage, but yet very dangerous misdiagnosis of a normal intrauterine pregnancy in cases of ectopic pregnancy.

**Table 1:** Demographic and clinical data of 106 women with suspected ectopic pregnancies

Variables	Number of records found N (%)	Mean ± SD	Range	
			Minimum	Maximum
Age (years)	(99%) 105	31.4 ± 5.6	20	44
Week of gestation	(93%) 99	6.2 ± 1.6	3	12
Beta-hCG level (mIU/ml)	(100%) 106	6,394 ± 22,988.4	5	159,000
Endometrial thickness (mm)	(81%) 86	11.75 ± 6	1	26

Abbreviation: SD, standard deviation.

As presented in Table 2, the sensitivity of frozen section analysis in the diagnosis of ectopic pregnancy was 72.7%, specificity 95.9%, positive predictive value 88.9% (24/27) and negative predictive value 88.6% (70/79) (Table 2). The median β-hCG level was lower for cases in which the frozen section diagnosis was accurate than for cases of inaccuracy (Table 3). This correlation was statically significant (*p* = 0.01). Endometrial thickness was greater for cases that showed discrepancy between frozen section analysis and pathological diagnosis; however, the difference was not statically significant (Table 4). More frozen section diagnoses were accurate in cases with preoperative serum hCG concentration < 1,000 mIU/ml than in cases with hCG concentration > 1,000 mIU/ml, *p* < 0.001 (Table 5). According to ROC: area under curve = 0.696, *p* = 0.001, confidence interval (CI) was 95% (0.583, 0.809). For β-hCG level = 1,000 mIU/ml, the sensitivity of accurate diagnosis was 61%, and the specificity was 70%.

**Table 2:** Results of frozen section and pathological examination

			Pathological diagnosis	
			Negative	Positive
Frozen section diagnosis	Negative	Number of cases	70	9
		%	95.9%	27.3%
	Positive	Number of cases	3	24
		%	4.1%	72.7%
Total		Number of cases	73	33
		%	100%	100%

**Table 3:** Beta-hCG level according to the accuracy of frozen section and pathological results

	Accuracy of FS and pathological diagnosis	Number of cases		Median	Minimum	Maximum	P¤
		Valid	Missing				
Beta-hCG level	Inaccurate	12	0	1,551.5	422	10,115	0.01
	Accurate	94	0	493.52	5	159,000	1-sided

Abbreviation: FS, Frozen section.

¤ *P*-value according to Wilcoxon rank sum test.

**Table 4:** Endometrial thickness according to the accuracy of frozen section and pathological results

	Accuracy of FS and pathological diagnosis	Number of cases			Minimum	Maximum	P¤
		Valid	Missing	Mean ± SD			
Endometrial thickness	Inaccurate	11	1	14.5 ± 5.4	8	25	0.101
	Accurate	75	19	11.3 ± 6	1	26	2-sided

Abbreviation: FS, Frozen section; SD, standard deviation.

¤ P-value according to Wilcoxon rank sum test.

**Table 5:** The accuracy of frozen section diagnosis according to preoperative serum hCG concentration: higher hCG concentration was associated with higher accurate diagnosis of the frozen section (*p* < 0.001)

hCG level (mIU/ml)	Number of cases	Diagnoses concur %	Diagnoses differ %
< 1000	64	60 93.7%	4 6.3%
> 1000	42	34 81%	8 19%

## Discussion

The management of PUL was thoroughly revised by Barnhart et al.^18^ In their review, several management protocols were suggested, including histologic confirmation of the diagnosis in some cases.^18^ Our study supports the use of frozen section technique as a rapid and accurate method of identifying products of conception on endometrial curettage. The 88.7% accuracy of frozen section analysis we reported is similar to that demonstrated by Barak et al.^10^ The positive and negative predictive values (88.9% and 88.6%, respectively), though high, are of limited strength due to the retrospective design of the analysis.

Of the incorrect diagnosis in our study, most were false negatives (9 of 106 cases), in which women were erroneously diagnosed by the frozen section technique with the absence of intrauterine pregnancy. In all nine cases, women started methotrexate therapy.

Although the single dose methotrexate protocol has fewer side-effects than the earlier multiple dose therapy, this antimetabolite treatment is not completely safe and has its drawbacks. Such drawbacks are bone marrow suppression and delaying the next pregnancy to ensure that the medication has been cleared from the organism, since methotrexate is associated with birth defects. Thus, methotrexate should be initiated only after the confirmation of a pathological diagnosis of ectopic pregnancy or after measuring β-hCG level at least once more after the procedure.

There were three false-positive diagnoses among our cohort, in which women were erroneously thought to have villi in the frozen section specimen and were thus misdiagnosed as not having an ectopic pregnancy. A possible explanation for this error is that part of a trophoblastic villus from the tubal pregnancy was shed into the uterus. As the amount of tissue may have been minimal, it could have been included in the frozen section specimen only, and not in the final pathology material. A false positive result poses the highest risk since a woman could be discharged from the hospital with an ectopic pregnancy that could rupture and be fatal. Therefore, even if the frozen section detects products of conception, and the woman is hemodynamically stable, a thorough monitoring of β-hCG should be done to ensure a downward trend. The occurrence of false positive and false negative results emphasizes the need to confirm the frozen section diagnosis with a definitive pathologic examination.

Beta-hCG was lower among those subjects with accurate frozen section diagnosis; this included both true positive (24 of 106) and true negative (70 of 106) cases. A possible explanation is that true negative cases comprise 75% of the accurate cases (70 of 94 cases); since this represents the absence of intrauterine pregnancy, β-hCG level is expected to be low. To improve the accuracy of the pregnancy location Seeber et al.^19^ suggested the use of a redefined hCG curves, and yet 12% of the patients with ectopic pregnancy were not diagnosed. The results of this study^19^ also emphasize the need to rely on more than one criterion to diagnose ectopic pregnancy, such as progesterone plasma concentrations.^20^

We initially hypothesized that greater endometrial thickness will have a significant correlation with frozen section diagnosis, because thickened endometrium will provide more material for frozen section evaluation. Our results did not show such relationship. We suggest, as an explanation for this result, that endometrial thickness, as measured by ultrasound, includes in most cases blood clots and not only products of conception.

The frozen section method provides a rapid means of diagnosing ectopic pregnancy. Advantages of this technique include early institution of therapy and reduction in the hospitalization period, with its inherent costs in cases of suspected abnormal intrauterine pregnancy. The patient can be discharged once the diagnosis of conception products is obtained by frozen section, and the uterus is evacuated. However, the adherence to strict criteria of diagnosing an abnormal intrauterine pregnancy or ectopic pregnancy is important to avoid the unintended termination of a wanted pregnancy.^21^

## Conclusion

In conclusion, frozen section examination is a rapid and mostly accurate method of diagnosing products of conception in endometrial curetting. We report a statically significant correlation between lower β-hCG levels and high accuracy of the frozen section technique. Such a correlation was not displayed between endometrial thickness and the accuracy of the frozen section technique. The results of our study suggest that implementation of this technique in the clinical management of an ectopic pregnancy enables more rapid diagnosis, facilitates earlier institution of treatment and shortens the period of hospitalization.
